# Anti-Inflammatory and Anti-Platelet Properties of Lipid Bioactives from Apple Cider By-Products

**DOI:** 10.3390/molecules26102869

**Published:** 2021-05-12

**Authors:** Alexandros Tsoupras, Donal Moran, Thomas Byrne, James Ryan, Luke Barrett, Con Traas, Ioannis Zabetakis

**Affiliations:** 1 Department of Biological Sciences, University of Limerick, V94 T9PX Limerick, Ireland; Donal.Moran@ul.ie (D.M.); tommyb1998@hotmail.com (T.B.); Jimmyryan1672@gmail.com (J.R.); luke.barrett@outlook.ie (L.B.); Con.Traas@ul.ie (C.T.); Ioannis.Zabetakis@ul.ie (I.Z.); 2 Health Research Institute, University of Limerick, V94 T9PX Limerick, Ireland; 3 Bernal Institute, University of Limerick, V94 T9PX Limerick, Ireland

**Keywords:** CVD, platelets, thrombosis, inflammation, polar lipids, apple pomace, PUFA, MUFA, PAF, ADP

## Abstract

The valorization of food industry by-products as sources of bioactive compounds is at the forefront of research in functional foods and nutraceuticals. This study focuses on bioactives of apple cider by-products (ACBPs) with putative cardio-protective properties. Total lipids (TLs) were extracted from ACBPs of apple varieties that are low (ACBP1), medium (ACBP2), and high (ACBP3) in tannins and were further separated into polar lipids (PLs) and neutral lipids (NLs). The functionality of these lipid extracts and of their HPLC-derived lipid fractions/PL subclasses were assessed in vitro against human platelet aggregation induced by the thrombotic and inflammatory platelet agonists platelet-activating factor (PAF) and adenosine diphosphate (ADP). The fatty acid profile of PLs and their most bioactive lipid fractions were evaluated by GC–MS analysis. The PL extracts exhibited higher specificity against the PAF-induced platelet aggregation compared to their anti-ADP effects, while TL and NL showed lower bioactivities in all ACBPs. HPLC analysis unveiled that the most bioactive PL from all ACBPs were those in PL fraction 3 containing phosphatidylcholines (PCs). PLs from all ACBPs and their PC bioactives were rich in polyunsaturated fatty acids (PUFAs) and especially in the essential omega-6 (n-6) linoleic acid (LA) and omega-3 (n-3) alpha linolenic acid (ALA), with favorably low values of the n-6/n-3 PUFA ratio, thus providing a rationale for their higher anti-inflammatory bioactivities. Within this study, highly bioactive PL compounds with strong anti-inflammatory and anti-platelet properties were identified in ACBPs, which can be potentially utilized for producing cardio-protective functional foods and/or nutraceuticals.

## 1. Introduction

Thrombotic and inflammatory complications have been connected to numerous chronic disorders, such as cardiovascular disease, diabetes, and cancer [1,2]. Inflammatory and thrombotic mediators, such as platelet-activating factor and thrombin, and several platelet agonists, such as ADP and collagen, are implicated in inappropriate leukocyte and platelet activation and aggregation as well as in endothelial dysfunction and the subsequent onset and development of the aforementioned inflammatory manifestations [1,2,3,4]. There is extensive research in the medical and pharmaceutical fields for producing several types of drugs against these pathways [4]. 

However, the exploitation of the benefits of healthy dietary interventions and of food bioactives and functional foods from sustainable dietary sources that can also prevent and protect against inflammatory processes and their related mediators involved in these pathologies has also gained interest as an alternative approach due to the lack of any unwanted side-effects [1,2,3,4,5]. Natural bioactive compounds found in fruit, vegetables, dairy fermented products and other beverages, oils, and meat, specifically fish, have become subject to research for their health benefits and wide array of uses in the health and wellbeing industry [1,2,5]. Moreover, the study of existence of bioactives in by-products of these food sources has also gained attention in the food industry in the last decades as a sustainable way of utilizing natural bioactives in functional foods and food supplements or nutraceuticals [6]. Bioactive lipid compounds and, more importantly, bio-functional dietary polar lipids (PLs) found in such foods and beverages have shown a significant impact in preventing/reducing the effects of thrombotic and inflammatory pathways involved in the onset and development of chronic disorders, including cardiovascular diseases (CVDs) [1,2,5,6]. 

According to the food and agriculture data of the food and agriculture organization of the united nations (FAOSTAT), apple is considered to be one of the most highly consumed fruits worldwide due to its nutritional value and being easily accessible throughout the year. Although apples and apple products are not a food source with high lipid content, the fruit and the food products (apple juice and cider) derived from it have recently been found to contain small amounts of bioactive PLs that have demonstrated similar anti-inflammatory and anti-platelet properties that have been shown in other healthy foods and beverages [7]. Nevertheless, during the processing of apples into apple products such as juice, cider, and vinegar, there is a large amount of waste product in the form of apple pomace. The worldwide apple industry produces several products such as juice, cider, and vinegar. This production leads to 70 million tons of waste which may be used as a functional product in other areas for its nutritional contents that have not been removed in the original processing [8]. Apple pomace has been subject to bioactive research in an effort to utilize the apple fruit for all its nutritional function ability. The literature shows compounds found in apples and apple products including pomace, such as lipids, polyphenols, and pectin, can also be extracted from the products to be used in other areas to increase the nutritional value and functionality of other foods/products [8,9,10].

Apple pomace is composed mainly of carbohydrates, fiber, protein, lipids, and ash [7,8,9,10,11,12,13,14]. These nutrients are primarily attributed to the skin and flesh (95%) [8]. Along with macronutrients, the apple is a source of phytochemicals such as phenolic compounds and flavonoids [8,9,10,11,12,13,14]. During processing, huge loss of many of these hydrophilic nutrients and bioactive compounds occurs due to their being transported to the water-based juice product. However, the skin left within the pomace also seems to contain phenolic compounds [8,9,10,11,12,13,14] and other bioactives in comparison to similar fruits such as grapes and grape pomace, where bioactives with anti-inflammatory properties against several pathways linked to inflammatory manifestations, including those of PAF-related complications, and against the subsequent oxidation of LDL-cholesterol have been reported [15,16]. 

Nevertheless, the hydrophilic compounds found in apples and in apple juice and their cider products have shown no significant effects against thromboinflammation and platelet aggregation induced either by PAF or ADP [7,17]. On the other hand, several hydrophilic/lipid bioactive compounds have recently been found in apple products such as apple juice and cider [7]. In both apple juice and apple cider derived from several apple varieties, the PLs and especially their subclasses of phosphatidylcholines (PCs) and phosphatidylethanolamines (PEs) exhibited potent anti-platelet and anti-inflammatory properties in human cells [7]. Nevertheless, apple cider by-products (ACBPs) from apple varieties used for producing apple juice/cider have not yet been studied for containing lipid bioactives with anti-inflammatory and anti-platelet benefits against the PAF and ADP pathways and platelet aggregation, providing a rationale for commencing an evaluation for these apple by-products too as sustainable sources of lipid bioactives. 

Thus, within the present study, the lipid content of ACBPs was evaluated for the first time as a potential source of lipid bioactives with putative anti-platelet and anti-inflammatory properties against the pathways of the inflammatory and thrombotic mediator PAF, but also against the pathways of a classic platelet agonist, ADP. The assessment of lipid composition and functionality in human platelets were performed for lipid bioactives extracted and separated from ACBPs of the Jonagold (low in tannins, ACBP1), Dabinett (intermediate in tannins, ACBP2), and Aston Bitter (high in tannins, ACBP3) apple varieties. Fractionation of ACBP lipids in bioactive subclasses by high-performance liquid chromatography (HPLC) and assessment of their fatty acid content by gas chromatography mass spectra (GC–MS) analysis were also utilized for evaluating structure–activity relationships in the lipid bioactives from all these ACBPs. Our firstly reported promising results may instigate the valorization of ACBP lipid bioactives in functional foods and nutraceutical industries. 

## 2. Results and Discussion

### 2.1. Yield Extraction of Lipids from ACBP and Fractionation by HPLC

The yields of extraction for the total lipids (TLs), neutral lipids (NLs), and PLs extracted from different ACBP samples are displayed in Table 1 and are expressed as % percentage for each sample (g of lipids extracted per 100 g of sample). Similarly to previous studies in apples and other food by-products [6,7], the Bligh and Dyer extraction process [18] in tandem with the Galanos and Kapoulas Counter Current Distribution technique [19] were also chosen in the present study to extract and separate TL, NL, and PL extracts from the apple pomace for each ACBP. When these two methods are combined, they allow a simple and effective extraction and separation approach for obtaining dietary polar lipid bioactives as has been shown in several solid or liquid natural sources [6,7,15,16,20,21,22,23]. They have also shown excellent outcomes with respect to the yield of extraction of such bioactive PLs in each of these natural sources, with their fatty acid composition also being intact [6,7,15,16,20,21,22,23]. Such an increased efficacy for obtaining bioactive PLs from natural sources by combining these two methods further ensures very low to no loss of the bioactivities of bio-functional lipids during their extraction process, unlike other methods such as Soxhlet extraction, that uses high temperature and may thus detrimentally affect the fatty acid content of the extracted lipids [24]. 

In addition, these methods were also chosen due to having been previously applied effectively for isolating and acquiring the bioactive PLs from the NLs from several similar plant-based sources and by-products for producing several beverages, such as apple cider, beer, wine, or even tea [7,15,16,21,22,23], in order to be able to observe their individual properties against inflammatory mediators in cell-models based bioassays. 

According to the results shown in Table 1, ACBP2 showed the highest yield for TL and PL which, however, was only statistically significant higher from that of ACBP3 that showed the lowest yield (*p* < 0.05 for these comparisons). By contrast, the yield of extracted lipids from both ACBP2 (the highest) and ACBP3 (the lowest) did not statistically differ significantly when compared with the relevant intermediate yield of ACBP1 (*p* > 0.05 for all these comparisons). Thus, even though the ACBPs assessed in this study originated from different apple sources varying in their tannin levels, this difference did not affect the yield of their TL, NL, and PL extracts.

In all ACBPs, the PLs made up approximately 55–85% of the TLs while the remaining 15–45% of the TLs seem to be its NL content, suggesting that the ACBPs of all these apple pomaces contain higher amounts of PL than NL. Nevertheless, it should also be stressed that according to the results shown in Table 1, ACBP1 was the only apple pomace assessed that exhibited statistically significant higher yield of its PLs when compared to the relative yield of its NLs (*p* < 0.05 for this comparison), suggesting that this variety contains, and thus can provide, more PLs than NLs, since approximately 75% of the TLs are PLs, while only the 25% of the TLs are NLs. 

Furthermore, the yield of TL and PL extraction for all these ACBPs were found to be of significantly higher yield (2–10 times higher) than the relevant yields of extraction for TL and PL from the relevant apple-derived juices and cider products for each type of low, medium, and high in tannin apple varieties (Jonagold, Dabinett and Aston Bitter, respectively) [7], but also when compared with the relevant yields for other beverages derived from plant/fruit sources, such as beer, wine, and tea [7,15,21,22,23]. Such relative differences were also observed in analysis of PLs in apples, in which 10 times more PLs were found in the apple skin (that usually remains mostly in the apple pomace after squeezing processing) in comparison to the PLs found in the apple flesh (that is usually squeeze-processed for apple juice and cider production) [25]. Thus, such results further suggest that the majority of the apple lipid content remains mainly in its apple pomace waste after processing for producing apple juice and cider products. 

Taking into account also that the yields of extraction for both TLs and PLs for all these ACBPs were similar to the yields of highly bioactive PLs found in other foods, such as several marine sources and their relevant by-products [6,20], further suggests that all these ACBPs and especially ACBP1 and ACBP2 are also good sources for acquiring bio-functional PLs. However more studies are needed, and especially in extraction procedures using environmentally friendly solvents and techniques, for fully evaluating the potential for high yield of food grade extracted bioactive PLs from each type of ACBP.

Since the PL extracts of all ACBPs showed potent anti-inflammatory and antithrombotic properties against PAF and anti-platelet effects against ADP, the polar compounds of these PL extracts from all ACBPs were further separated into six main fractions/PL subclasses by HPLC analysis, as previously described [7]. For all these PL extracts, HPLC analysis was performed using a wavelength of 208 nm, where double bonds in lipids and, thus, the lipids themselves can be detected, but also at 280 nm, where phenolic groups usually are detected. The individual fractions represented the respective subclasses of polar compounds within the PL extracts by utilizing specific standards (Table 2).

**Figure 1:**
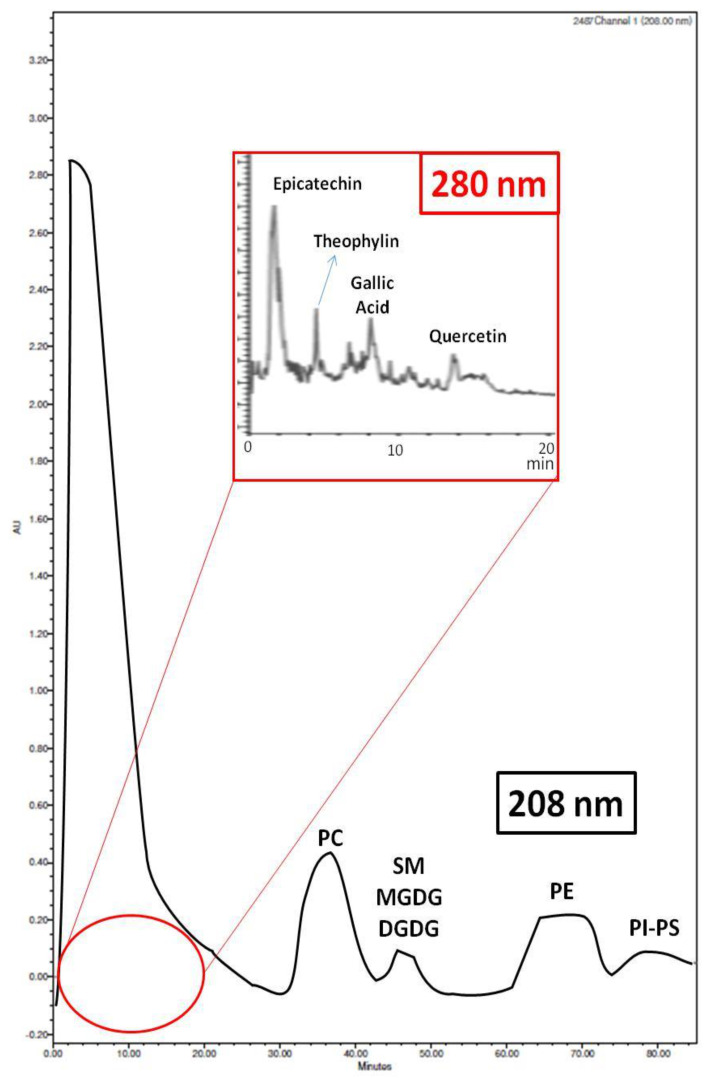
Representative chromatogram of HPLC analysis of ACBPs. Labeling of peaks was based on the use of specific standards and analysis in both 208 nm (for polar lipids) and 280 nm (for phenolic compounds). Abbreviations: PC, phosphatidylcholine; SM, sphingomyelin; PE, phosphatidylethanolamine; PI, phosphatidylinositol; PS, phosphatidylserine; MGDG, monogalactosyldiglyceride; DGDG, digalactosyldiglyceride; ACBP, apple cider by-product.

More specifically, the separation of the six PL fractions shown in Table 2 of the PL extracts for each one of the ACBP was performed according to their retention times in comparison with the retention times obtained for several standards of phenolic compounds (detected at 280 nm) and PL molecules (specific glycerol/sphingosine-based phospholipids, glycolipids, and sulfatides/sulfolipids), detected at 208 nm, by applying a specific gradient mobile phase in a normal phase column for this HPLC analysis, which has been previously described in detail during a similar HPLC analysis of the PL extracts obtained from the apple juices and cider products from the same apple varieties (Jonagold, Dabinett and Aston Bitter, respectively) [7]. A representative chromatogram of such analysis is shown in Figure 1.

According to this HPLC analysis and based on the retention times of the relative standards analyzed, as shown in Table 2, fraction 1 compounds comprising of molecules with phenolic groups were eluted at 0–15 min, polar sphingo-based glycolipid compounds were eluted in fraction 2 at 15–30 min, PC molecules of PLs were eluted in fraction 3 at 30–45 min, sphingomyelin (SM) and some sulfo- and glycerol-based glycolipid compounds such as monogalactosyldiglyceride (MGDG) and digalactosyldiglyceride (DGDG) were eluted in fraction 4 at 45–60 min, while fraction 5 contained PE molecules of PLs that where eluted at 60–75 min, and the remaining fraction 6 was comprised of phosphatidylinositol (PI), phosphatidylserine (PS), and of remnants of other more polar lipid compounds such as phosphatidylglycerol (PG), eluted at 75–90 min. 

The presence of such PL subclasses in apple pomace observed in the present study is also in accordance with previously reported outcomes in apple skin and apple flesh [25], but also in apple juice and cider [7]. From weighing the PL fractions obtained from such HPLC analysis of 1 mg of the PL extracts from all ACBP, very similar percentage values for each fraction were observed between the different ACBP apple pomaces, with the exception of fraction 1 that contains phenolics, the percentage of which was increased from ACBP1 (low in tannins) to ACBP3 (high in tannins). More specifically, approximately 35–45% of the PLs in all apple pomaces (ACBP1, ACBP2, and ACBP3) was found to be PC, 20–30% was PE, 15–25% was MGDG, DGDG, and all the other lipids eluted in fraction 4 such as SM and sulfatides, while only 10–25% were phenolics eluted in fraction 1 (approximately 10% in ACBP1, 10–20% in ACBP2, and 20–25% in ACBP3) and 5–15% PI, PS, and other polar lipid compounds like PG that were eluted in fraction 6 as well as 5–10% of lipids eluted in fraction 2. Such relative % composition of the PL fractions within the PL extracts observed in all apple pomaces of the present study are similar to the ones previously observed in both apple skin and flesh [25]. However, more sophisticated lipidomic approaches, based on modern MALDI-TOF LC–MS, are needed for quantifying and structurally elucidating all PL bioactive molecules present in each PL subclass from the PLs of all ACBPs to support the above findings, as was previously described for the grape pomace and Irish ale beer cases [16,21].

Furthermore, all TL, NL, and PL extracts for each ACBP apple pomace and each of the six HPLC-derived PL fractions were further assessed in the platelet aggregometry bioassay for their ability to inhibit platelet aggregation induced either by the PAF-related inflammatory and thrombotic pathways or by the well-established platelet activation agonist, ADP (for PL), while the fatty acid composition of all PL extracts and of the most bioactive HPLC-derived PL subclasses were further elucidated by GC–MS analysis. 

### 2.2. Anti-Inflammatory and Anti-Platelet Properties of the ACBP Lipid Extracts (TL, NL, and PL) and HPLC-Derived Bioactive PL Subclasses

**Figure 2:**
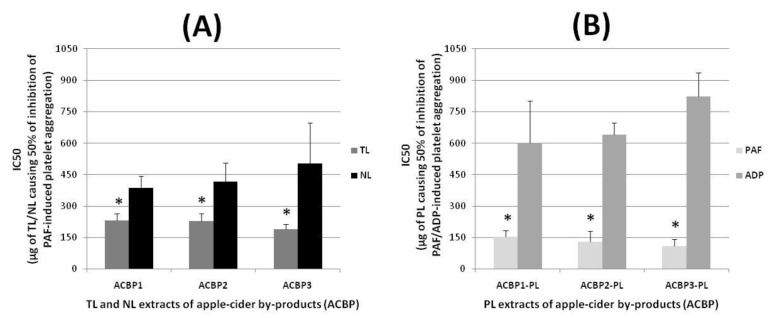
The anti-inflammatory and anti-platelet potency of TL (**A**), NL (**A**), and PL (**B**) extracts from ACBP, against human platelet aggregation induced by the inflammatory and thrombotic mediator PAF (for TL, NL, and PL) or by the platelet agonist ADP (for PL). Results are expressed as means of the IC_50_ (half-maximal inhibitory concentrations) values in µg of TL, NL, and PL in the aggregometer cuvette that causes 50% inhibition of PAF/ADP-induced platelet aggregation (the lower the IC_50_ value for a lipid extract the higher its inhibitory effect against the specific agonist of platelet aggregation). * denotes statistically significant difference (*p* < 0.05) when the anti-PAF potency (IC_50_ value) of the bioactive TL extracts were compared with the relative anti-PAF potency of the NL extracts for each ACBP (**A**), or when the anti-PAF potency (IC_50_ value) of the bioactive PL extracts were compared with their relevant anti-ADP potency for each ACBP (**B**). Abbreviations: TL, total lipids; NL, neutral lipids; PL, polar lipids; ACBP, apple cider by-products (apple pomace); ACBP1, apple cider by-products of low in tannin Jonagold apple variety; ACBP2, apple cider by-products of medium in tannin Dabinett apple variety; ACBP3, apple cider by-products of high in tannin Aston Bitter apple variety; PAF, platelet-activating factor; ADP, adenosine 5′ diphosphate.

The biological activities of the TL, NL, and PL extracts from the three types of apple pomaces, ACBP1, ACBP2, and ACBP3, were evaluated by acquiring their putative anti-inflammatory and anti-platelet potency against human platelet activation and aggregation induced by the inflammatory and thrombotic mediator PAF as previously described [6,7,20,23]. The results obtained from these bioassays in human platelets are shown in Figure 2, in which the anti-inflammatory and antithrombotic potency of the bioactive ACBP lipid extracts (TL, NL, and PL) are expressed as means of their IC_50_ (half-maximal inhibitory concentrations) values in µg of ACBP TLs, NLs, and PLs in the aggregometer cuvette that causes 50% inhibition of PAF-induced platelet aggregation. It should also be stressed that the lower the IC_50_ value for a lipid bioactive against the PAF-induced platelet aggregation, the more superior its inhibitory effect against the PAF pathways of inflammation and thrombosis.

All lipid extracts exhibited potent anti-inflammatory and antithrombotic activities against the PAF pathway of aggregation of human platelets, with the PLs in each ACBP sample showing the most potent inhibitory effects against the PAF pathway of platelet aggregation, with IC_50_ values of their anti-PAF activities being approximately within the range of 70–180 μg (Figure 2B), which were found to be statistically significant lower (and thus more bioactive) than the relative anti-PAF IC_50_ values of both TLs and NLs for each ACBP shown in Figure 2A (*p* < 0.05 for all these comparisons). On the other hand, the NL extracts of all ACBP samples assessed showed the lowest inhibition against the PAF pathway of platelet aggregation, with IC_50_ values of these anti-PAF effects being approximately within the range of 300–700 μg (Figure 2A), which were found to be statistically significant higher (and thus less bioactive) than the relative IC_50_ values of both PLs and TLs for each ACBP (*p* < 0.05 for all these comparisons). Thus, the TL extracts in all ACBP samples showed intermediate but considerable potency against PAF-induced human platelet aggregation, with the IC_50_ values of these inhibitory effects against the PAF pathway being approximately within the range of 180–270 μg (Figure 2A). The intermediate inhibitory action observed for the TL extracts of each one of the three ACBPs against PAF-induced platelet aggregation seem to be derived by the combination of their more-active PL content and less-active NL content.

These results are in accordance with previously reported ones for similar differences observed in the anti-inflammatory properties against the PAF pathway of human platelet aggregation between the TL, NL, and PL extracts of apple products (apple juice and cider) [7] which were produced from the low (Jonagold), medium (Dabinett), and high (Aston Bitter) in tannin apple varieties that are relevant to the ACBP apple varieties assessed, and were extracted and separated with the same methodology applied in the present study. More specifically, in both the apple juice and cider products of these three apple varieties, the PL extracts were found again to be more bioactive against PAF [7], as in the case of their relative ACBP apple pomaces observed in the present study. In addition, the differences observed in these anti-PAF effects between the TL, NL, and PL extracts in each ACBP sample are also in accordance with previously reported similar differences for the anti-PAF bioactivities of the TL, NL, and PL extracts of other beverages and plant/fruit-derived sources and by-products for beverage production, such as wine, beer, and tea [15,16,21,22,23], which were also extracted and separated with the same methodology applied in the present study.

It should also be stressed that no statistically significant difference was observed when the anti-PAF potency (IC_50_ values) of the PL extracts from ACBP1 were compared to the relevant ones for the PL extracts of the medium in tannin ACBP2 and the high in tannin ACBP3 (*p* > 0.05 in all these comparisons of the different PL bioactivities). This result further suggests that the potent anti-inflammatory and anti-thrombotic potency of the PL extracts in all ACBP apple pomaces seem to not be associated to their tannin content. Similarly, no differences were observed when comparing the anti-PAF bioactivities of all TL extracts from these three ACBPs within each other (*p* > 0.05 in all these comparisons of the different TL bioactivities), or even when comparing the NL anti-PAF effects from these three ACBPs (again *p* > 0.05 in all these comparisons of the different NL bioactivities), which further support the notion that the observed anti-PAF bioactivities of the ACBP lipid extracts is irrelevant to their tannin content. This finding may also be associated to a possible migration and loss of tannins (as more hydrophilic compounds) at the hydroalcoholic phase during the Bligh and Dyer extraction process. However, this assumption requires further research in order to be confirmed, and especially such comparisons in food grade lipid extracts of ACBP with different tannin contents using environmentally friendly solvents and food grade approaches.

Nevertheless, the PL from all ACBP were found to be similar to less active and within the same order of magnitude as the PAF-associated inflammatory and thrombotic pathways when compared to the anti-PAF bioactivities of the PL from their relative apple products (apple juice and cider) [7], which were also produced from the same apple varieties used for the ACBP and were also extracted and separated with the same methodology applied in the present study and also from other beverages and plant/fruit-derived sources and by-products for beverage production, such as grapes, wine, yeasts, and winery by-products (grape pomace) [15,16], beer and brewery by-products [21,22], tea [23], and olive pomace [26].

Since the PLs were the lipid compounds in all three ACBP apple pomaces with the most potent anti-PAF effects in human platelets, and in order to evaluate their overall putative anti-platelet beneficial properties, the ACBP-derived PL extracts were also further assessed against the platelet aggregation induced by a classic and well-established platelet agonist, ADP, which activates platelets through pathways other than those of platelet aggregation induced by PAF [1,2,3,4], as previously described [6,7,23]. The results obtained from these bioassays in human platelets are also shown in Figure 2B, while the anti-platelet potency of the ACBP PL bioactive extracts were again expressed as means of their IC_50_ values (µg of ACBP PL in the aggregometer cuvette that causes 50% inhibition of ADP-induced platelet aggregation). Once more, the lower the IC_50_ value for a lipid bioactive against ADP-induced platelet aggregation, the more superior its inhibitory effect against the ADP-associated thrombotic pathways.

The PL extracts from each ACBP showed considerable but significantly lower anti-platelet properties against the ADP pathway of platelet aggregation, with higher IC_50_ values for their anti-ADP activities that were within the range of 300–700 μg (Figure 2B), which were also found to be statistically significant higher (and thus less bioactive) than the relative anti-PAF potency (IC_50_ values) of these PL extracts in each ACBP (*p* < 0.05 for all these comparisons). These results are in accordance with previously reported ones observed in dietary PLs and further suggest that the bioactive PL extracts of ACBP apple pomaces also have higher specificity against the PAF-associated inflammatory and thrombotic pathways rather than against other platelet-activation pathways induced by classic platelet agonists, such as ADP. Similar superior anti-PAF properties of several dietary PLs, such as the aforementioned ones observed for the PL extracts of all ACBP apple pomaces assessed in the present study, seem to be associated with their structural resemblance to PAF and the subsequent structure–activity relationships of their antagonistic effects against the binding of PAF to the unique for PAF G-coupled protein cell membrane receptor (PAF-R) [1,2,3,4,5,6,7,15,16,20,21,22,23,26].

Such an observed higher efficacy of the PL extract from the ACBP1 apple pomace, low in tannin, against the inflammatory PAF pathway in comparison to its lower anti-ADP effects has not been previously observed in bioactive PLs from apple products (apple juices and cider products) from the same apple variety (Jonagold) [7]. In contrast, in both apple juice and cider from apples low in tannin, the anti-PAF efficacy of their PL was similar to their efficacy against the ADP pathway [7]. This outcome further suggests that some PL bioactives of the low in tannin apple variety (Jonagold), that possess strong anti-ADP efficacy, seem to migrate to the apple products (apple juice and cider) during processing and, to a lesser extent, within the relevant ACBP remnants/wastes of such processing. Nevertheless, the anti-PAF activities of the PL extracts from both the apple products (apple juice and cider) and their ACBP of the low in tannin apple variety (Jonagold), were of similar potency (within the same order of magnitude), suggesting that the PL bioactives of this apple variety with strong anti-PAF efficacy seem to migrate equally to its apple products (apple juice and cider) and to the relevant ACBP remnants/wastes during processing.

Moreover, no statistically significant difference was observed between these low anti-ADP effects of the PL from these ACBP apple pomaces, which further support the notion that apart from the anti-inflammatory potency against PAF, the anti-platelet potency of the bioactive ACBP lipids against the ADP-pathway is also not associated with their tannin content.

**Figure 3:**
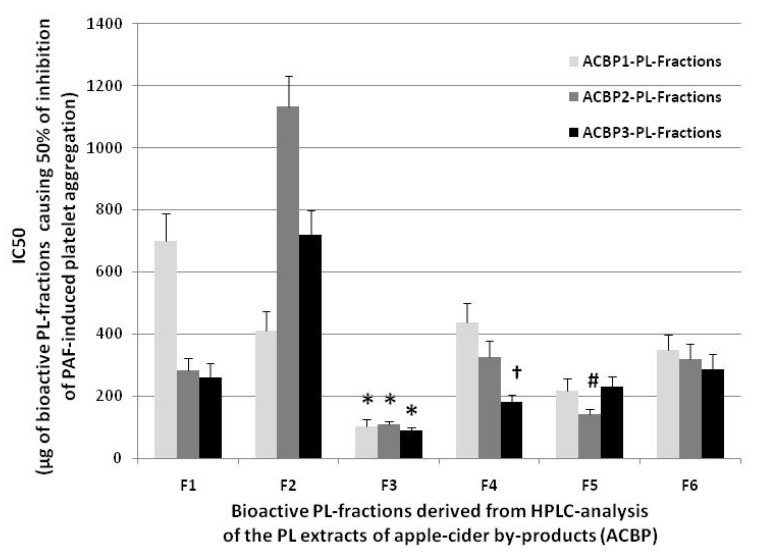
The anti-inflammatory potency of the bioactive HPLC-derived PL subclasses for each ACBP against human platelet aggregation induced by the inflammatory and thrombotic mediator PAF. Results are expressed as means of the IC_50_ (half-maximal inhibitory concentrations) values in µg of the bioactive PL compounds of each PL fraction in the aggregometer cuvette that causes 50% inhibition of PAF-induced platelet aggregation (the lower the IC_50_ value for a bioactive lipid fraction, the higher its inhibitory effect against the PAF-associated inflammatory pathways). * denotes statistically significant difference (*p* < 0.05) when the anti-PAF potency (IC_50_ value) of the bioactive PL fraction 3 (F3) corresponding to the PL subclass containing bioactive PC molecules were compared with the relative anti-PAF potency of all the other PL fractions for each ACBP apple pomace. “#” denotes statistically significant difference (*p* < 0.05) when the anti-PAF potency (IC_50_ value) of the bioactive PL fraction 5 (F5) of the ACBP2, corresponding to the PL subclass containing bioactive PE molecules, were compared with the relative anti-PAF potency of the same PL fraction F5 of the other two ACBP1 and ACBP3 apple pomaces, as well as with all the other PL fractions for each ACBP (apart from the F4 of ACBP3). “†” denotes statistically significant difference (*p* < 0.05) when the anti-PAF potency (IC_50_ value) of the bioactive PL fraction 4 (F4) of the ACBP3, corresponding to the PL subclass containing bioactive SM and several glycolipid molecules were compared with the relative anti-PAF potency of the same PL fraction F4 of the other two ACBP1 and ACBP2 apple pomaces, as well as with all the other PL fractions for each ACBP (apart from the F4 for all ACBP). Abbreviations: PL, polar lipids; F, PL fraction corresponding to specific PL subclass that were derived by HPLC analysis of the PL extracts for each ACBP; ACBP, apple cider by-products (apple pomace); ACBP1, apple cider by-products of low in tannin Jonagold apple variety; ACBP2, apple cider by-products of medium in tannin Dabinett apple variety; ACBP3, apple cider by-products of high in tannin Aston Bitter apple variety; PAF, platelet-activating factor.

Since the PL extracts of all assessed ACBPs were the most bioactive against the inflammatory PAF pathway, as mentioned earlier, they were further separated into molecular subclass fractions using HPLC analysis, performed using a wavelength of 208 nm, where double bonds in lipids and, thus, the lipids themselves can be detected, but also at 280 nm, where phenolic groups are usually detected. All the PL subclasses/fractions obtained from the HPLC analysis of each ACBP PL extract were also assessed for their putative bioactivities to inhibit the PAF-induced aggregation of human platelets. The results obtained from these bioassays in human platelets are shown in Figure 3, in which the anti-inflammatory potency of the ACBP-derived bioactive PL subclasses are again expressed as the means of their IC_50_ values (µg of ACBP-derived bioactive PL compound in the aggregometer cuvette that causes 50% inhibition of PAF-induced platelet aggregation). Similarly, the lower the IC_50_ value for a lipid bioactive compound (PL subclass) against the PAF-induced platelet aggregation, the more superior its inhibitory effect against the PAF-associated inflammatory and thrombotic pathways.

Differently than the overall PL extracts that showed potent anti-inflammatory properties against the PAF pathway for all ACBPs (Figure 2), the anti-inflammatory potency for all the HPLC-derived ACBP PL fractions (IC_50_ values shown in Figure 3) varied depending on the different PL subclasses present in each PL fraction. Within all these assessed PL fractions, lipid fraction 3 (F3), in which the bioactive PC molecules were eluted, exhibited the most potent anti-PAF effects when compared to all the other PL fractions in all ACBP apple pomaces (*p* < 0.05 for all these comparisons). These results are in accordance with those previously reported for the relative lipid fraction 3 containing PC molecules of the PLs from apple juice, which were derived from the same apple varieties (Jonagold, Dabinett and Aston Bitter) by following similar experimental methodology [7], further suggesting that the PC bioactives of these apple varieties with strong anti-PAF efficacy seem to migrate equally to its apple juice product and to the relevant ACBP apple pomace remnants/wastes during processing.

In addition, these results are also in accordance with those observed in relevant studies in other healthy foods such as oily fish (salmon) [20], beverages such as beer [21], and other dietary sources such as microorganisms of biotechnological interest in the food industry, such as microalgae (*Spirulina*) [27] and bioethanol-producing bacteria (*Zymomonas mobilis*) [28], in which, again, the PL fractions containing bioactive lipid molecules belonging to the PC family were the most potent anti-inflammatory lipid subclass against the PAF pathway in several models of inflammation and platelet aggregation [1,20,21,27,28]. It should also be stressed that such dietary bioactive PC molecules are constitutionally more abundant in small, dense HDL cholesterol, where they preferably bind, while these small, dense HDL lipids enhance HDL functionality and increase HDL levels, embodying cardio-protective properties, antithrombotic potency, and antioxidant protection in cells and against LDL oxidation [1,29].

Moreover, the PL subclass corresponding to the bioactive PE molecules that were eluted in lipid fraction 5 (F5) also showed an intermediate and considerably potent anti-inflammatory bioactivity against the PAF pathway, which was statistically less potent than the anti-PAF effects of the relative PC molecules, but more potent than all the other lipid fractions in all ACBPs, with the exception of the lipid fraction 4 (F4) for the ACBP3 that also showed a considerable strong anti-PAF bioactivity. Similar dietary PE bioactives from other food sources, such as those eluted in lipid fraction F5 of the ACBPs, were also reported to possess strong anti-PAF effects in several cells, including platelets [1,20,21]. It should be mentioned that within the PL fraction F4, bioactive MGDG, DGDG, SM and some sulfatides are usually eluted, which were also previously observed in Irish ale beer with strong anti-platelet effects, while they have also shown strong anti-inflammatory and antitumor properties [21].

On the contrary, very low anti-PAF potency was found in the lipid fraction 2 (F2) in which sphingo-based glycolipids were eluted in all ACBP, and especially in ACBP2, that exhibited the lowest anti-PAF effects. These results suggest that such sphingoglycolipid bioactives seem to be less bioactive or in lower amounts in all ACBPs in comparison to all the other lipid bioactives eluted in the other lipid fractions of ACBP PLs.

Furthermore, in all ACBPs, the lipid fraction 6 (F6), which contains the more polar PLs (i.e., PIs, PSs, etc.), was found to have stronger anti-PAF bioactivities than the lipid molecules of F2. In contrast, PLs in F6 were less bioactive against the PAF pathway when compared to either the PC fraction F3 or the PE fraction F5. Nevertheless, the anti-PAF effects of the PLs in F6 were comparable to those found in F4 which, as aforementioned, contains SM, glycerol-based glycolipids such as MGDG and DGDG, and sulfatides only in ACBP1 and ACBP2. These bioactivities in both F6 and F4 were also similar to the anti-PAF effects found in fraction 1 (F1), which contains phenolic compounds/functional groups only in the case of ACBP2 and ACBP3. Exceptions to the above are F4 from the PLs of ACBP3 which, as aforementioned, exhibited stronger anti-PAF effects comparable to those of the PE molecules of F5, but also the phenolics of F1 in the low in tannin ACBP1 that showed very low anti-PAF potency.

Interestingly, apart from F1 of ACBP1, the stronger anti-PAF bioactivities observed in the phenolics eluted in fraction 1 of both ACBP2 and ACBP3 were found to be approximately 3 times more potent than those previously reported for the relative apple juices from intermediate and high in tannin Dabinett and Aston Bitter apple varieties, respectively. This result seems to be associated with the higher content of tannins in these apple varieties as mirrored by the relatively higher % composition of F1 in ACBP2 and ACBP3 in comparison to that of the low in phenolic content of F1 from the low in tannin ACBP1, indicating that some more bioactive phenolics remain mainly in the apple pomace after processing for apple juice and cider production, especially in the cases of ACBP2 and ACBP3. However, more studies are needed to fully elucidate the relative anti-inflammatory and anti-platelet contribution of the phenolics eluted in F1 of the PL from apple pomace.

Overall, the strong anti-inflammatory potency observed in the PL extracts of all ACBP apple pomaces against the PAF pathway seem to be derived by the interplay of all these lipid bioactives eluted in their HPLC-derived lipid fractions which, on some occasions, have also been found to act synergistically against PAF-induced inflammatory platelet aggregation, leukocyte activation, endothelial dysfunction, and LDL oxidation, thus reducing the risk for CVD and other inflammation related chronic disorders [1]. Nevertheless, more studies are needed in order to elucidate such potential for the ACBP lipid bioactives that will further enhance the use of apple pomaces as a sustainable source for such PL bioactives.

### 2.3. Fatty Acid Composition of Bioactive PL from ACBP by GC–MS

The fatty acid composition of the PL extracts and of their most bioactive PC subclass from all ACBP apple pomaces was elucidated by GC–MS analysis, and the results are displayed in Table 3 and Table 4, respectively. All PL extracts from these three ACBPs were found to be rich in polyunsaturated fatty acids (PUFAs), followed by lower amounts of saturated fatty acids (SFA) and the less abundant monounsaturated fatty acids (MUFA) (Table 3). More specifically, all bioactive ACBP-derived PL extracts contained high amounts of the most abundant essential omega-6 (n-6) PUFA, linoleic acid (LA) (C18:2 c9, 12 n-6), followed by the essential omega-3 (n-3) PUFA, alpha linolenic acid (ALA) (C18:3 c9,12,15 n-3), and by much less but considerable amounts of other bioactive n-3 PUFAs such as eicosapentaenoic acid (EPA) (C20:5 c5,8,11,14,17 n-3), docosapentaenoic acid (DPA) (C22:5 c7,10,13,16,19 n-3), and docosahexaenoic acid (DHA) (C22:6 c4,7,10,13,16,19 n-3). In all PL extracts of the three ACBPs, hexadecanoic acid (C16:0) was the most abundant SFA, while the most abundant MUFA was oleic acid (C18:1 c9), but in considerably lower amounts than the aforementioned SFA and PUFA content of PL from ACBP. Similar fatty acid compositions were also obtained from the GC–MS analysis of the most bioactive PC lipid fractions of these PL extracts in all ACBPs (Table 4).

These results are in accordance with the previously reported fatty acid content of the bioactive PL extracts of apple products (apple juice and cider) from the same apple varieties (Jonagold, Dabinett, Aston Bitter) and of the PC bioactives of the PL extract of apple juices from the low in tannin Jonagold apple variety [7], and in apples in general [25], while they again further suggest that such PL bioactives rich in n-3 PUFA seem to migrate equally to its apple juice/cider products and to the relevant ACBP apple pomace remnants/wastes during processing.

Interestingly, it has been previously proposed that plant sources do not contain the long chain n-3 PUFA EPA, DPA, and DHA due to lack of appropriate enzyme machinery for producing them from ALA and LA, yet Guil et al. have reported the presence of low amounts of both EPA and DHA in several natural plants [30], which was also recently observed in tea PL bioactives [23] but also in the ACBP-derived PL bioactives assessed in the present study and in the PL bioactives of apple products (apple juice and cider) from the same apple varieties (Jonagold, Dabinett, and Aston Bitter) [7], while apple-derived rhamnogalacturonan II, the most structurally complex segment of the 10% of apple pectin, and its unusual sugar-based compounds have also been reported to contain DHA as determined by GC–MS of their trimethylsilyl-esters O-methyl glycosides [31].

In addition, the presence of such essential n-3 PUFA (mostly ALA, but also much lower but considerable amounts of EPA, DPA, and DHA) bound in the bioactive PL extracts of the ACBP, and especially in their most potent PC bioactives, further support their anti-inflammatory potency and provide a rationale for their strong anti-PAF effects as previously described in several other bioactive dietary PLs and PCs of natural origin, and especially in PL bioactives from healthy food sources [1,6,7,20,21,22,23]. Such dietary PLs rich in n-3 PUFA have been found to inhibit platelet aggregation induced by the inflammatory and thrombotic mediators, PAF and thrombin, but also by classic well-established platelet agonists such as collagen and ADP [1,6,7,20,21,22,23] as was also observed in the present study for the rich in n-3 PUFA bioactive PL extracts of ACBPs and their potent PC bioactives. Nevertheless, apart from the bioactivities observed on the PL bioactives, the n-3 PUFA content of these PLs and especially of the PC bioactives has, on its own, several beneficial bio-functionalities, especially when released from these PL in cells by specific cytoplasmic phospholipases A2 (PLA2) enzymatic activities. For example, the PLA2-based release of n-3 PUFA from such bioactive PLs in cell membranes and/or lipoproteins facilitate the production of anti-inflammatory eicosanoids that act antagonistically to other inflammatory and thrombotic eicosanoids (prostaglandins, leukotrienes, and thromboxanes) usually produced by n-6 PUFA such as arachidonic acid and LA [32]. The latter further supports the health benefits derived from the aforementioned n-3 PUFA (ALA, EPA, DPA, and DHA), while healthy dietary patterns based on these n-3 PUFA have shown strong preventative benefits against several chronic disorders, such as in a Mediterranean diet enriched in ALA for the secondary prevention of coronary heart disease [33].

Subsequently, a relative index of the anti-inflammatory potency of dietary lipid bioactives, such as the bioactive PLs rich in n-3 PUFA that are present in healthy foods and diets, is the n-6/n-3 PUFA ratio, for which it has been proposed that the lower the value for this ratio, the better the preventative anti-inflammatory benefits against several inflammation- and platelet aggregation-related chronic disorders, and vice versa [32]. In the PL extracts of all ACBPs, the n-6 PUFA content was not so much higher than their n-3 PUFA content, which resulted in favorable low values of the n-6/n-3 PUFA ratio of these bioactive ACBP PLs within the range of 1.4–2.9 (Table 3), while for the PC bioactives, this ratio ranged at much lower values of approximately 1.1–2.0 (Table 4) that were lower than previously reported values for this ratio in apples [34] and apple juices [7]. The low values of the n-6/n-3 PUFA ratio observed in ACBP-derived PL bioactives and especially in their PC bioactives are usually observed in healthy foods and diets, while they are also much lower than the values above 15/1 for this ratio that are usually observed in unfavorable Western-style/Westernized foods and diets [32]. The above findings of the favorable low values for the n-6/n-3 PUFA ratios in PL bioactives of all the tested ACBPs, and especially in their bioactive PC subclasses, further support the potential anti-inflammatory and cardio-protective properties of the ACBP-derived PL bioactives.

## 3. Materials and Methods

### 3.1. Materials, Reagents, and Instrumentation

Platelet aggregation materials for use in the bioassays were purchased from Labmedics LLP (Abingdon on Thames, UK). Safety needles (20G) and evacuated sodium citrate S-monovettes for blood sampling were from Sarstedt Ltd. (Wexford, Ireland). The standards PAF and bovine serum albumin (BSA) were acquired from Sigma Aldrich (Wicklow, Ireland), and Chronolog (Havertown, PA, USA) supplied the ADP for bioassays. Chronolog-490 two channel turbidimetric platelet aggregometer (Havertown, PA, USA) coupled to the accompanying AGGRO/LINK software package was used for the analyses of human platelet-rich plasma (hPRP) for platelet aggregation bioassays. The Eppendorf 5702R centrifuge (Eppendorf Ltd., Stevenage, UK), was used for the centrifugations. Quartz 1 cm cuvettes were used within the Shimadzu UV-1800 spectrophotometer (Kyoto, Japan) for spectrophotometric analysis.

HPLC analysis was carried out using the Alliance e2695 Separations Module in tandem with a Waters 2487 UV detector and an Empower Chromatography Data Software was applied for the separation of apple pomace bioactive PLs into subclasses. GC–MS experimentation was performed using a Varian 410-Gas Chromatographer coupled to a Varian 210-MS detector equipped with a split/splitless injector (Agilent Technologies, Palo Alto, CA, USA). The standards and reagents used for GC–MS and HPLC were supplied by Sigma Aldrich (Wicklow, Ireland). All additional glass and plastic consumables, solvents, and reagents were of analytical grade and were purchased from Fisher Scientific Ltd. (Dublin, Ireland). Flash rotary evaporation (Buchi Rotavapor, Mason Technology Ltd., Dublin, Ireland) was used for the evaporation of solvents from all lipid extracts, while nitrogen stream from nitrogen cylinders (BOC, Dublin, Ireland) was used for evaporations in a nitrogen environment.

### 3.2. ACBP Apple Pomace Samples

This experiment required three samples for our analysis. “Con Traas’s Apple Farm” provided bags of apple pomace which were sourced from the plantation located in Co. Tipperary. The apples used in production were all grown according to normal commercial practice on this apple farm site in County Tipperary. The farm provided 3 varieties of apple pomace including Jonagold (low in tannins), Dabinett (intermediate tannin content) and Aston Bitter (high in tannins). The apples are washed and then sent to a pressing machine at the farm. The juice was then pressed from the apples and sent for further clarification and pasteurization or fermentation. The ACBP that remains after the pressing stage is also known as the “apple pomace”. This by-product or the pulp comprises the core, exhausted soft tissue, peel, stems, and seeds. Three samples (*n* = 3) of 100 g from each one of these three types of ACBPs (low, intermediate, and high in tannins) were assessed in the present study.

### 3.3. Extraction and HPLC Fractionation of Lipid Bioactives from ACBP

TL were extracted from different (*n* = 3) 100 g samples of each type of ACBP using the Bligh and Dyer extraction method [18] and further separated into NLs and PLs by the Galanos and Kapoulas counter current distribution technique [19] as previously described [7,20]. More specifically, TL extraction is achieved, as previously described [7,20], by homogenization of the sample in a monophasic system containing chloroform/methanol/water at a 1:2:0.8 (*v*/*v*/*v*) ratio, and then by filtrating the extracts from the precipitated remnants with filtering papers of 110 mm (Whatman, Maidstone, UK) under vacuum conditions by pumping in a Buchner-based filtering device. The homogenate/filtrate is then transferred to a separatory funnel and addition of appropriate volumes of water and chloroform is then performed in order to adjust the chloroform/methanol/water-based homogenate to a ratio of 1:1:0.9 (*v*/*v*/*v*) to achieve phase separation with the TL being present in the lower phase. Then, the NLs and PLs were separated by the Galanos and Kapoulas counter current distribution methodology [19] as previously described in the case of apple juice [7].

All the extracted lipid samples were collected in round bottom flasks and further evaporated until all solvents and water content were removed on a flash rotary evaporator at 37 °C under vacuum between 700 and 40 mbar (Buchi Rotavapor, Mason Technology Ltd., Dublin, Ireland), and then re-dissolved in a chloroform/methanol solution at a ratio of 1/1 (*v/v*) and transferred to a small pre-weighed glass tube, which was evaporated under nitrogen stream. The obtained TLs, NLs, and PLs were then weighed and stored under nitrogen at −20 °C for a maximum of 8 weeks before further analysis.

All high-performance liquid chromatography analysis of the PLs from each ACBP were performed as previously described [7].

### 3.4. Platelet Aggregometry Bioassays

The evaluation of the anti-inflammatory and anti-platelet properties of all TL, PL, and NL extracts from all ACBP samples, as well as of the bioactive lipid fractions derived from their HPLC analysis, were performed in human platelet-rich plasma (hPRP) preparations from healthy donors (*n* = 6) by assessing their ability to inhibit the aggregation of human platelets induced by the inflammatory and thrombotic mediator PAF and by the well-established platelet agonist ADP, as previously described [20,23,35]. The anti-inflammatory and antithrombotic potency of the bioactive ACBP-derived TL, NL, and PL extracts and of their PC-bioactives were expressed as means of their IC_50_ (half-maximal inhibitory concentrations) values ± standard deviation (SD), presented in mass (µg) of the bioactive lipid compound in the aggregometer cuvette that causes 50% inhibition of PAF/ADP-induced platelet aggregation, as previously described [7,20,23,35]. For ensuring reproducibility, these experiments were performed several times in different volunteer’s blood samples for each lipid bioactives in all ACBP samples (*n* = 6).

### 3.5. Gas Chromatography Mass Spectrometry (GC–MS)

Preparation and analysis of the fatty acid methyl esters (FAME) for all apple by-product lipid samples (PL extracts and the PC bioactive lipid fractions derived from their HPLC analysis) were carried out as previously described [20].

### 3.6. Statistical Analysis

Kolmogorov–Smirnov criterion was used to test normality of the IC_50_ values and fatty acid composition obtained for each lipid sample from ACBP. Subsequently, for comparisons of the lipid content and FA composition of the PLs from ACBP, acquired from the GC–MS analysis, the Kruskal–Wallis nonparametric multiple comparison test was used, while one-way analysis of variance (ANOVA) was used for all comparisons of IC_50_ values of these lipid compounds against ADP- and PAF-induced platelet aggregation. The differences were statistically significant when the *p*-values were less than 0.05 (*p* < 0.05). Analysis of the data was carried out using a statistical software package (IBM-SPSS statistics 26 for Windows, SPSS Inc., Chicago, IL, USA).

## 4. Conclusions

Within the present study, the presence of bioactive PL extracts and compounds such as PC, PE, and several glycolipids was identified for the first time in wastes/by-products produced during processing of apple-related products (apple juice and cider), such as the ACBP apple pomace. These ACBP-derived PL bioactives were found to possess strong anti-inflammatory properties, mainly against the inflammatory and thrombotic mediator PAF, but also considerable anti-platelet benefits against the pathways of other well-established classic platelet agonists, such as ADP, in human platelets. In addition, their high n-3 PUFA content and the favorable low values of their n-6/n-3 PUFA ratio further support their anti-inflammatory potency.

Finally, ACBP-derived bioactive PL compounds had similar anti-inflammatory potency in each type of ACBP from apple varieties of low, medium, and high tannin contents that were assessed, while almost one order of magnitude higher yield of PLs was achieved compared to the previously reported yield for PLs from apple juice and cider. These outcomes may emphasize the potential use of ACBP apple pomaces as sustainable sources for not only flavonoid phytochemicals and pectin bioactives but also for PL bioactives with anti-inflammatory and anti-platelet potential that can be used for fortification of other foods and in food supplements and nutraceuticals. Although these results are promising, further research and analysis is needed in order to fully evaluate the potential benefits and utilization of these PL bioactives found in ACBP apple pomaces.

## Figures and Tables

**Table 1:** Yield of TL, NL, and PL extracts from apple cider by-products ACBP1, ACBP2, and ACBP3, expressed as g/100 g ^1^.

Samples	TL ^1^	NL ^1^	PL ^1^
ACBP1	0.33 ± 0.17	0.08 ± 0.02	0.25 ± 0.12 ^#^
ACBP2	0.62 ± 0.25 *	0.20 ± 0.1	0.42 ± 0.2 *
ACBP3	0.16 ± 0.06	0.07 ± 0.35	0.09 ± 0.04

^1^ expressed as mean ± SD (*n* = 3); * statistically significant difference between the yield of TL and PL of ACBP2 when compared to those of ACBP3, respectively (*p* < 0.05 for all these comparisons); ^#^ statistically significant difference between the yield of PL when compared to that for NL in ACBP1 (*p* < 0.05 for this comparison). Abbreviations: TL, total lipid; NL, neutral lipid; PL, polar lipid; ACBP, apple cider by-product.

**Table 2:** Fractionation of the PL ^1^ extracts for each ACBP ^1^ into HPLC fractions/PL subclasses with relative retention times, according to specific standards.

HPLC-Fraction (PL Subclass)	Retention Time (min)	Relative Standards Used (Concentration—Retention Time)	Wavelength (nm) of Observed Detection
Fraction 1 (Phenolic compounds)	0–15	Epicatechin (3 mg/mL—2–4 min) Theophylline (3 mg/mL—3–5 min) Gallic Acid (3 mg/mL—5–8 min) Quercetin (3 mg/mL—12–15 min)	280 nm
Fraction 2 (Sphingo-based glycolipids)	15–30	Cerebrosides (0.5 mg/mL—25–30)	208 nm
Fraction 3 (PC ^1^)	30–45	L-α-Phosphatidylcholine (1.5 mg/mL—35–40 min)	208 nm
Fraction 4 (Lyso-PC, sphingo-based phospholipids, glycerol-based glycolipids such as MGDG and DGDG ^1^, sulfoglycolipids)	45–60	Sphingomyelin (0.3 mg/mL—45–50 min) L-α-Lysophosphatidylcholine (0.3 mg/mL—50–53 min) Digalactosyldiglyceride (0.3 mg/mL—53–56 min) Sulfatide (0.3 mg/mL—56–59 min)	208 nm
Fraction 5 (PE ^1^)	60–75	L-α-Phosphatidylethanolamine (1.2 mg/mL—62–67 min)	208 nm
Fraction 6 (PI, PS, and other more polar compounds)	75–90	L-α-Phosphatidylinositol sodium salt (0.9 mg/mL—75–78 min)	208 nm

^1^ Abbreviations: PL, polar lipid; ACBP, apple cider by-product; HPLC, high-performance liquid chromatography; PC, phosphatidylcholine; PE, phosphatidylethanolamine; PI, phosphatidylinositol; PS, phosphatidylserine; MGDG, monogalactosyldiglyceride; DGDG, digalactosyldiglyceride.

**Table 3:** The fatty acid profile of the PL extracts for each ACBP apple pomace, expressed for each FA as the mean value of its % percentage in the total fatty acids of each sample assessed (mean ± standard deviation (SD), *n* = 3).

Fatty Acid	PL Extracts of ACBP1	PL Extracts of ACBP2	PL Extracts of ACBP3
C12:0	0.06 ± 0.01	ND	ND
c14:0	0.68 ± 0.158	0.22 ± 0.16	0.19 ± 0.005
C15:0	0.15 ± 0.03	ND	0.12 ± 0.003
C16:0	17.69 ± 0.64	19.47 ± 0.55	18.30 ± 1.48
C16:1 c9	0.23 ± 0.03	0.07 ± 0.13	0.14 ± 0.04
C17:0	0.46 ± 0.04	0.28 ± 0.02	0.56 ± 0.04
C18:0	7.79 ± 0.29	6.17 ± 0.47	6.58 ± 0.20
C18:1 c9	6.45 ± 0.48	7.78 ± 0.99	6.54 ± 0.53
C18:2 c9,12 (LA)	45.79 ± 1.13	40.30 ± 3.14	38.51 ± 2.40
C18:3 c9,12,15 (ALA)	13.81 ± 0.45	11.65 ± 0.16	20.68 ± 2.56
C20:0	1.79 ± 0.47	3.71 ± 0.50	2.89 ± 0.39
C20:1 c11	0.18 ± 0.07	0.80 ± 0.13	0.36 ± 0.26
C20:2 c11,14	0.47 ± 0.19	0.63 ± 0.37	0.38 ± 0.25
C20:3 c8,11,14	0.10 ± 0.03	0.95 ± 0.74	0.517 ± 0.45
C20:4 c5,8,11,14	0.22 ± 0.08	1.19 ± 0.84	0.74 ± 0.91
C20:4 c8,11,14,17	0.27± 0.13	0.56 ± 0.46	0.39 ± 0.17
C20:5 c5,8,11,14,17 (EPA)	1.89 ± 0.08	2.83 ± 2.71	1.56 ± 1.01
C22:1 c13	0.56 ± 0.16	0.87 ± 0.78	0.42 ± 0.53
C22:5 c7,10,13,16,19 (DPA)	1.08 ± 0.41	1.65 ± 0.56	0.64 ± 0.18
C22:6 c4,7,10,13,16,19 (DHA)	0.37 ± 0.17	0.99 ± 0.34	0.61 ± 0.07
SFA	28.57 ± 0.96	29.76 ± 1.56	28.54 ± 1.48
MUFA	7.42 ± 0.59	9.51 ± 0.69	7.46 ± 0.36
PUFA	64.01 ± 0.40	60.73 ± 1.83	64.01 ± 1.14
n-6	46.59 ± 1.11	43.06 ± 1.32	40.14 ± 2.26
n-3	17.42 ± 0.71	17.66 ± 2.81	23.87 ± 2.98
n-6/n-3	2.68 ± 0.17	2.48 ± 0.47	1.71 ± 0.31

Abbreviations: PL, polar lipids; ACBP, apple cider by-products (apple pomace); ACBP1, apple cider by-products of low in tannin Jonagold apple variety; ACBP2, apple cider by-products of medium in tannin Dabinett apple variety; ACBP3, apple cider by-products of high in tannin Aston Bitter apple variety; n-3, omega-3 PUFA; n-6, omega-6 PUFA; PUFA, polyunsaturated fatty acids; MUFA, monounsaturated fatty acids; SFA, saturated fatty acids; ALA, alpha linolenic acid; LA, linoleic acid; EPA, eicosapentaenoic acid; DPA, docosapentaenoic acid; DHA, docosahexaenoic acid; ND, non-detectable.

**Table 4:** The fatty acid profile of PL fraction 3 containing the PC bioactives of each ACBP apple pomace, expressed for each FA as the mean value of its % percentage in the total fatty acids of each sample assessed (mean ± standard deviation (SD), *n* = 3).

Fatty Acid	PL Fraction of PC Bioactives from ACBP1	PL Fraction of PC Bioactives from ACBP2	PL Fraction of PC Bioactives from ACBP3
C12:0	0.03 ± 0.002	ND	ND
c14:0	0.54 ± 0.13	0.13 ± 0.158	0.09 ± 0.01
C15:0	0.15 ± 0.03	ND	0.06 ± 0.01
C16:0	18.43 ± 1.19	17.40 ± 0.78	18.14 ± 1.18
C16:1 c9	0.33 ± 0.15	0.32 ± 0.17	0.18 ± 0.04
C17:0	0.46 ± 0.04	0.44 ± 0.07	0.27 ± 0.11
C18:0	7.47 ± 0.35	7.75 ± 0.68	8.44 ± 0.95
C18:1 c9	6.68 ± 0.82	6.50 ± 0.95	6.59 ± 0.64
C18:2 c9,12 (LA)	36.11 ± 2.02	42.73 ± 0.60	38.11 ± 3.96
C18:3 c9,12,15 (ALA)	23.99 ± 1.65	20.24 ± 0.83	22.72 ± 2.19
C20:0	1.59 ± 0.29	1.62 ± 0.30	1.79 ± 0.14
C20:1 c11	0.22 ± 0.02	0.19 ± 0.08	0.15 ± 0.04
C20:2 c11,14	0.07 ± 0.02	0.07 ± 0.02	0.13 ± 0.02
C20:3 c8,11,14	0.05 ± 0.03	0.07 ± 0.03	0.15 ± 0.03
C20:4 c5,8,11,14	0.07 ± 0.02	0.08 ± 0.02	0.20 ± 0.04
C20:4 c8,11,14,17	0.33 ± 0.08	0.29 ± 0.04	0.24 ± 0.11
C20:5 c5,8,11,14,17 (EPA)	1.20 ± 0.10	0.75 ± 0.14	0.68 ± 0.19
C22:1 c13	0.56 ± 0.16	0.32 ± 0.10	0.82 ± 0.17
C22:5 c7,10,13,16,19 (DPA)	0.54 ± 0.08	0.34 ± 0.12	0.34 ± 0.07
C22:6 c4,7,10,13,16,19 (DHA)	1.21 ± 0.11	0.81 ± 0.15	0.95 ± 0.11
SFA	28.65 ± 1.48	27.30 ± 0.54	28.73 ± 1.87
MUFA	7.79 ± 1.09	7.32 ± 1.18	7.75 ± 0.55
PUFA	63.56 ± 0.55	65.38 ± 0.65	63.52 ± 2.32
n-6	36.29 ± 2.04	42.95 ± 0.58	38.59 ± 3.91
n-3	27.27 ± 1.74	22.43 ± 1.16	24.93 ± 2.46
n-6/n-3	1.34 ± 0.16	1.92 ± 0.13	1.57 ± 0.31

Abbreviations: PL, polar lipids; PC, phosphatidylcholine; ACBP, apple cider by-products (apple pomace); ACBP1, apple cider by-products of low in tannin Jonagold apple variety; ACBP2, apple cider by-products of medium in tannin Dabinett apple variety; ACBP3, apple cider by-products of high in tannin Aston Bitter apple variety; n-3, omega-3 PUFA; n-6, omega-6 PUFA; PUFA, polyunsaturated fatty acids; MUFA, monounsaturated fatty acids; SFA, saturated fatty acids; ALA, alpha linolenic acid; LA, linoleic acid; EPA, eicosapentaenoic acid; DPA, docosapentaenoic acid; DHA, docosahexaenoic acid; ND, non-detectable.

## Sample Availability

Not available.
